# Incidental Diagnosis of Complete Agenesis of the Corpus Callosum in a 62-Year-Old Man Presenting With Dizziness

**DOI:** 10.7759/cureus.107232

**Published:** 2026-04-17

**Authors:** Leana J Pande, Kyle Sharron, Damin Singh, Gabrielle Brini, Scott Woolf

**Affiliations:** 1 Neurology, Garnet Health Medical Center, Middletown, USA; 2 Neurology, Touro College of Osteopathic Medicine, Middletown, USA

**Keywords:** acute dizziness, corpus callosum agenesis, intellectual disabilities, mri brain scan, neuroimaging studies

## Abstract

Agenesis of the corpus callosum (ACC) is a congenital malformation most commonly identified in prenatal or early childhood imaging, with adult diagnoses being relatively rare. We present the case of a 62-year-old man with a history of intellectual disability who was incidentally found to have complete ACC during evaluation for dizziness and chest discomfort. Initial noncontrast computed tomography (CT) of the brain demonstrated ventricular enlargement concerning for hydrocephalus, prompting further evaluation. Subsequent magnetic resonance imaging (MRI) clarified the diagnosis, revealing classic radiographic features of ACC, including colpocephaly, the “moose head” configuration on coronal views, “racing car ventricles” on axial imaging, and the “sunray” (or “sunrise”) sign on sagittal views. The patient had no prior neuroimaging and was previously unaware of this congenital anomaly. This case highlights the potential for delayed diagnosis of ACC in individuals with lifelong intellectual disability and emphasizes the importance of recognizing characteristic imaging findings to avoid misinterpretation. Additionally, it underscores the clinical and social relevance of identifying structural brain abnormalities later in life.

## Introduction

Agenesis of the corpus callosum (ACC) is a congenital malformation defined by partial or complete absence of the corpus callosum, critical for interhemispheric integration [1,2]. The corpus callosum is the largest commissural fiber bundle in the brain and plays a central role in the transfer of sensory, motor, and cognitive information between hemispheres [2]. Current population estimates suggest a prevalence of approximately 0.5 to 70 in 10,000, and approximately 25% of individuals diagnosed with isolated ACC postnatally have demonstrated intellectual disability [1,2]. The estimate of prevalence varies widely, and it is thought to be overdiagnosed. The clinical presentation of ACC is variable and may range from asymptomatic individuals to those with developmental delay, seizures, cognitive impairment, and neuropsychiatric symptoms, depending on whether the anomaly is isolated or associated with other structural abnormalities [2,3].

ACC results from the disruption of commissural development between approximately 8 and 20 weeks of gestation, leading to partial or complete absence of callosal fibers [2,3]. There is no specific treatment for ACC itself, and management is typically supportive, focusing on associated neurologic, developmental, or psychiatric conditions when present [2]. In clinical practice, diagnosis is most often made prenatally, initially detected by routine ultrasound or in early childhood when imaging is obtained for developmental concerns or abnormal neurologic findings [1,4]. Current American Academy of Pediatrics (AAP) guidelines recommend neuroimaging in children with intellectual disability primarily when additional clinical features are present, such as abnormal head circumference, focal neurologic deficits, seizures, or regression [4]. Consequently, individuals who do not meet these imaging thresholds in childhood, particularly those with mild or nonspecific developmental findings, may never receive neuroimaging during early life. Given this association, ACC can remain undiagnosed into adulthood.

While ACC is well characterized in prenatal and pediatric populations, late-in-life diagnoses and radiographic signs are scarcely discussed in the literature. Most reported cases are identified early in life due to developmental delay, seizures, or associated anomalies, whereas incidental discovery in older adults remains uncommon. Several adult case reports have documented incidental ACC identified in the fifth decade of life or later, underscoring that this diagnosis, while rare, is not confined to childhood [5,6]. Barriers to diagnosis in adulthood include lack of prior neuroimaging, attribution of cognitive findings to baseline intellectual disability rather than to an underlying structural etiology, and misinterpretation of ventricular enlargement on initial imaging [3,7].

Here, we present a case of incidentally diagnosed complete ACC in a 62-year-old man during evaluation for dizziness, highlighting both the classic imaging findings and the potential for delayed recognition in patients with longstanding intellectual disability. The delay in this patient’s diagnosis likely reflects two compounding factors: the absence of routine prenatal ultrasound screening, which was not widely implemented until the 1970s, along with the limited availability of clinical MRI during his early life, and the contemporary tendency to attribute cognitive findings to an assumed baseline rather than pursue structural investigation [8]. Characteristic MRI findings include the “moose head” configuration and “racing car ventricles” on coronal and axial views, as well as the “sunrise” sign on sagittal imaging.

## Case presentation

A 62-year-old male patient with a medical history of hypertension, angina, orthostatic hypotension, and intellectual disability presented to the emergency department with dizziness and chest discomfort. While the patient was at work, he suddenly developed lightheadedness and a room-spinning sensation. On arrival, vital signs were within normal limits; however, orthostatic measurements were positive. His dizziness improved with intravenous hydration and administration of meclizine. Cardiology was consulted, and hydrochlorothiazide was discontinued, given concern for medication-related orthostasis.

On physical examination, the patient was alert and oriented to person and place, consistent with his baseline. Cranial nerve examination was intact. Motor strength was 5/5 in all extremities without focal deficits. Sensory examination was normal to light touch. Coordination testing, including heel-to-shin, was intact, and pronator drift was negative. Speech was fluent without dysarthria.

**Figure 1:**
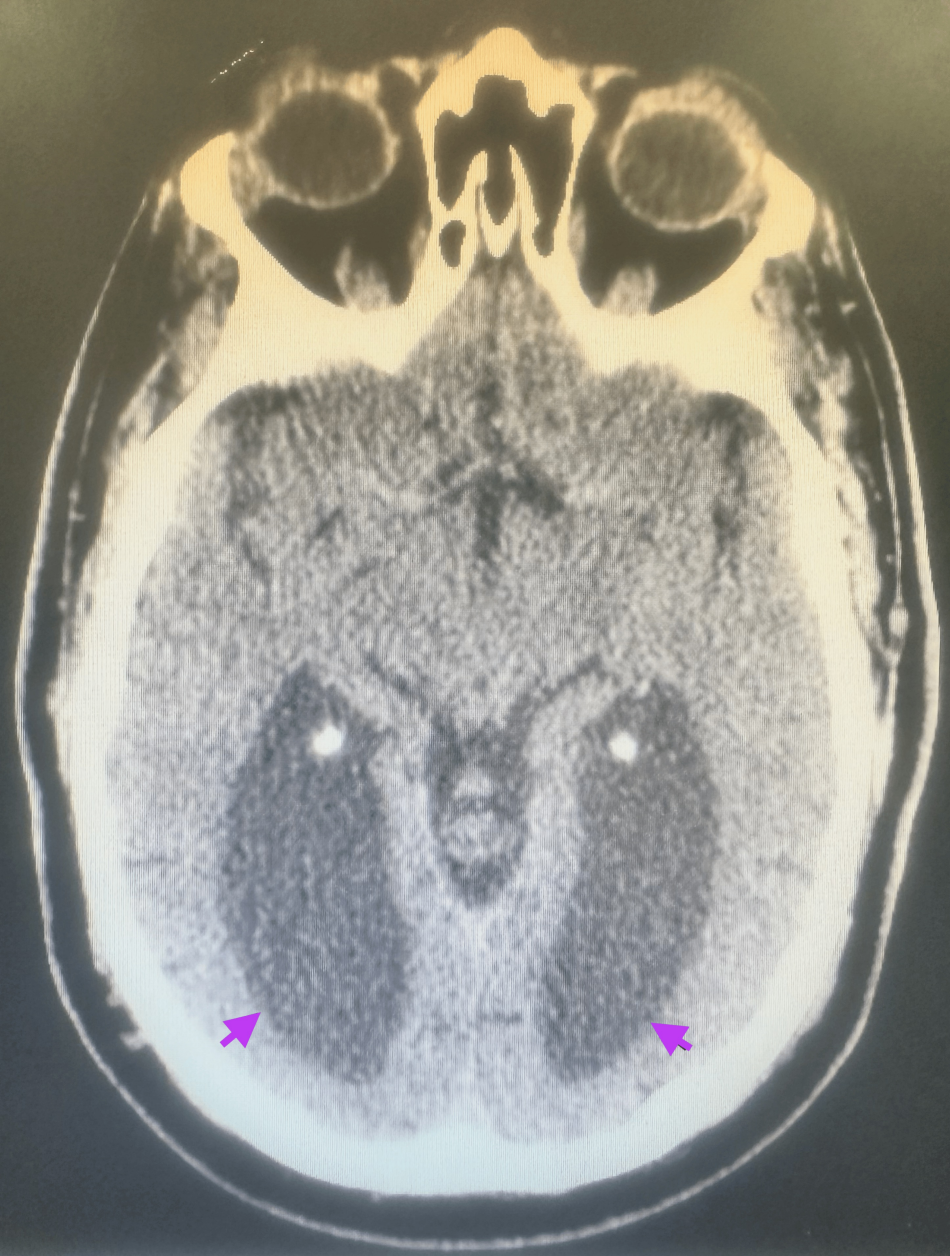
CT showing enlarged ventricles.

**Figure 2:**
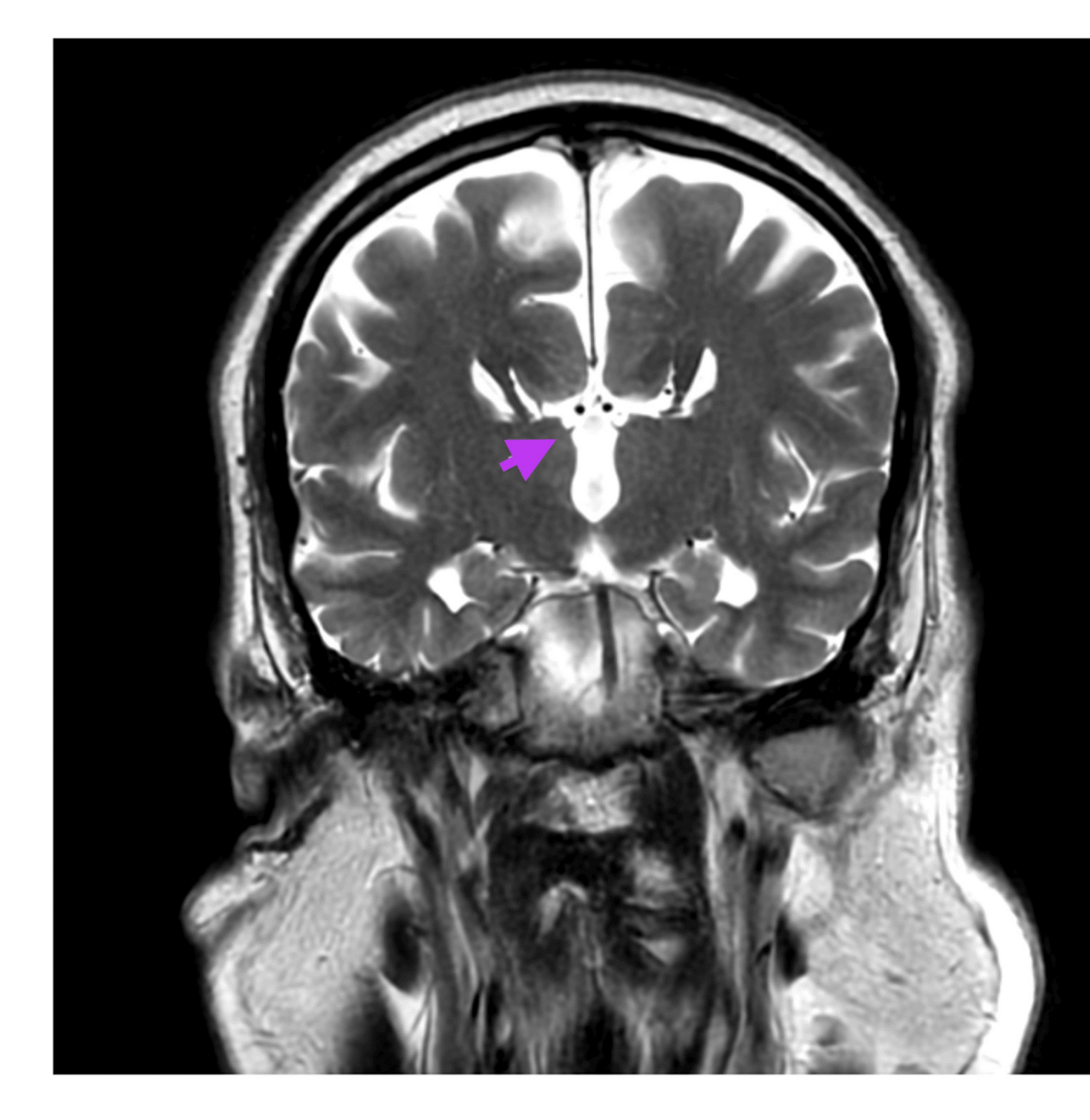
T2 propeller MRI brain coronal view displaying the “moose” sign characteristic of corpus callosum agenesis.

**Figure 3:**
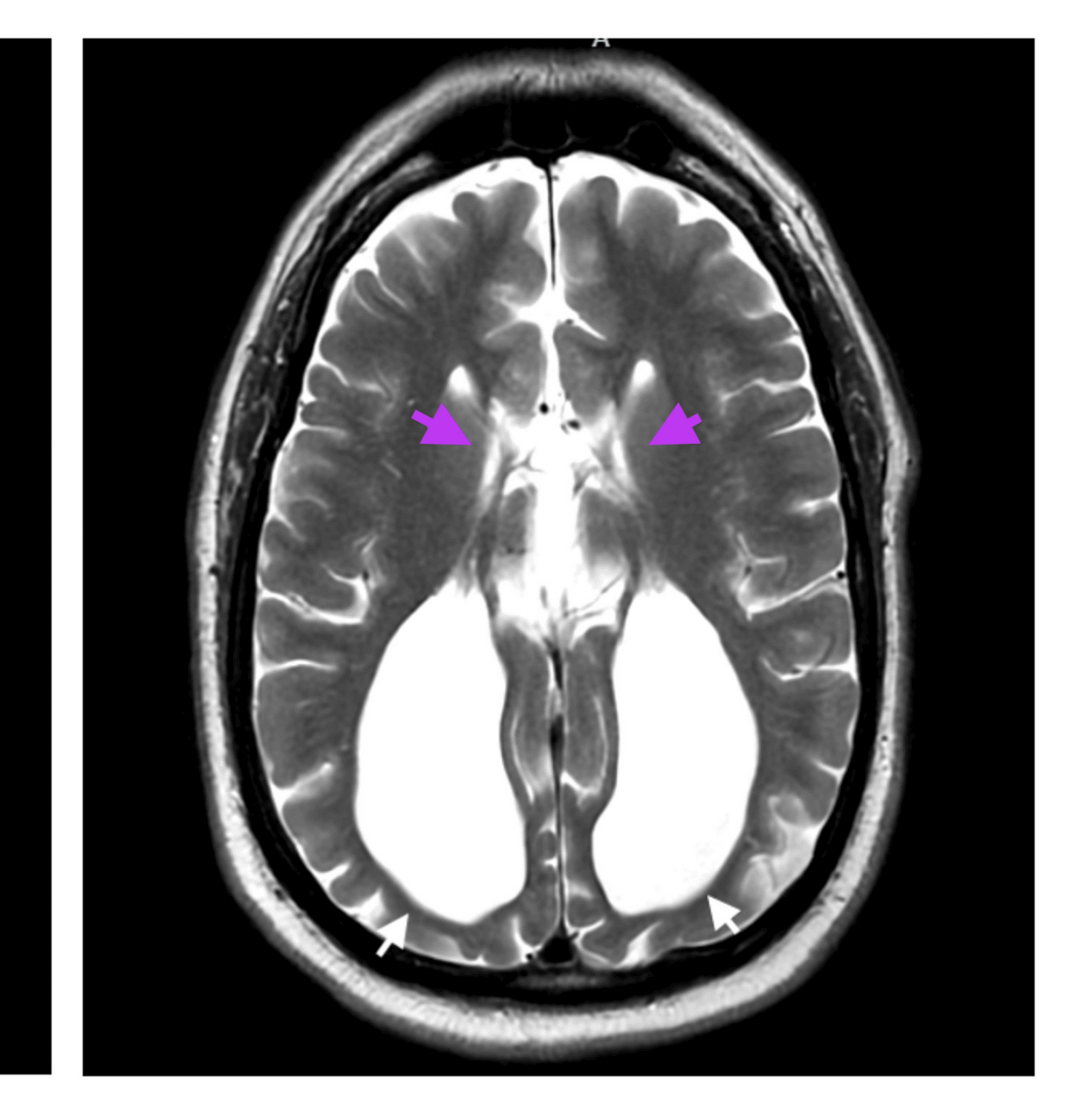
T2 propeller MRI brain axial view displaying the “racing car” sign characteristic of corpus callosum agenesis.

**Figure 4:**
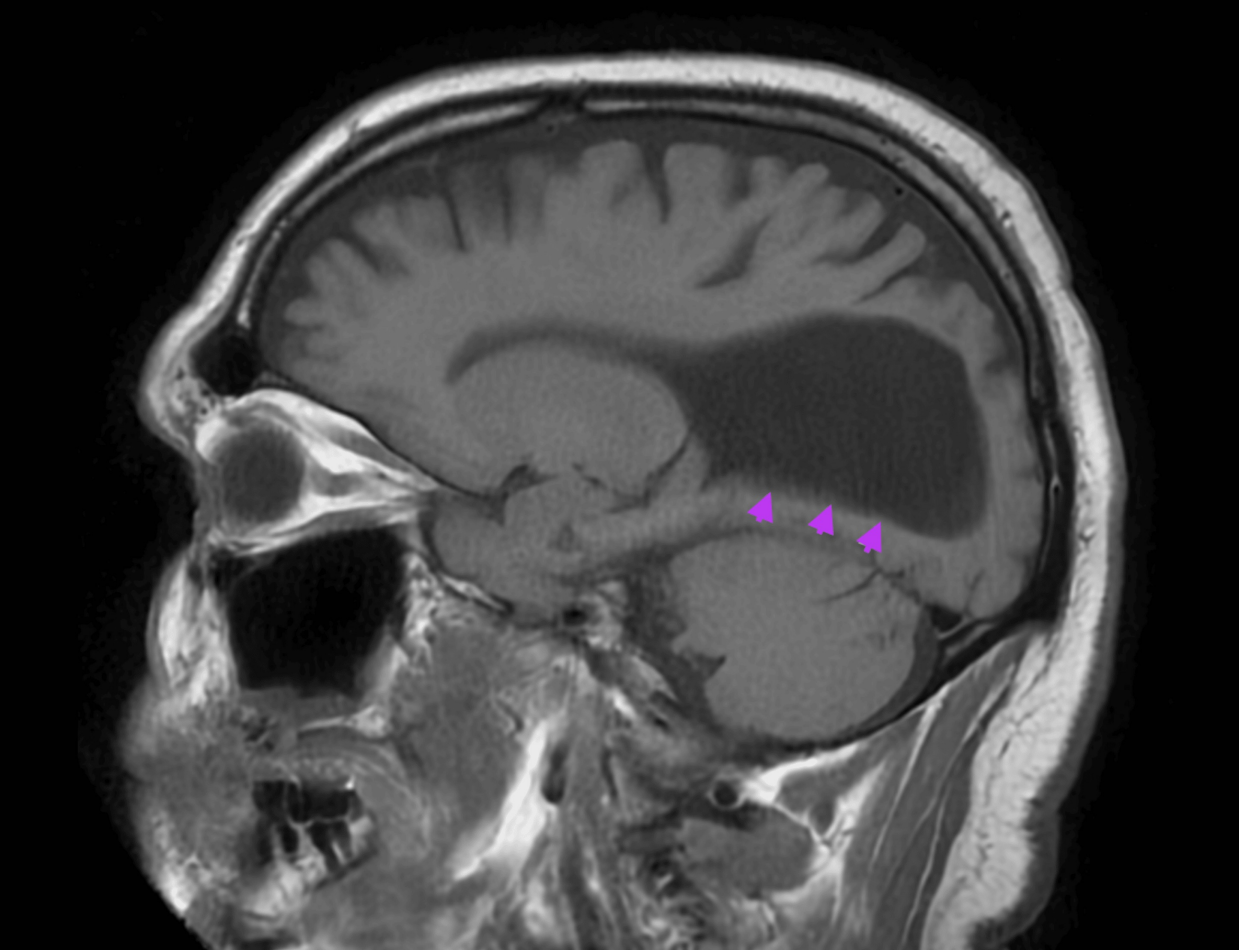
T1 MRI brain sagittal view of the “sunrise” sign characteristic of corpus callosum agenesis.

A noncontrast CT of the brain obtained in the emergency setting demonstrated enlarged ventricles and rounded frontal horns, which prompted neurology consultation due to concern for hydrocephalus from the primary team (Figure 1). Subsequent magnetic resonance imaging (MRI) of the brain clarified the initial diagnosis of “hydrocephalus” and instead demonstrated ACC. MRI revealed complete ACC with associated colpocephaly, including the “moose head” appearance and “racing car ventricles” (Figures 2, 3), as well as the “sunrise” sign on sagittal views (Figure 4). No additional intracranial abnormalities or associated malformations were identified on CT or MRI.

This finding was previously unknown to the patient. He reported a history of intellectual disability since childhood and participation in special education but had no prior knowledge of any structural brain abnormality. Discussion with the patient’s collateral sources and review of prior medical records confirmed a history of intellectual disability since childhood with no prior neuroimaging or known structural brain abnormality. The imaging findings were incidental and not related to his presenting symptoms, although they demonstrated classic features of corpus callosum agenesis on both CT and MRI. The patient required no acute neurologic intervention and was returned to the care of the medicine team. At the time of discharge, the patient was not established with outpatient neurology and was given a referral for follow-up. The patient has not followed up with Neurology since discharge.

## Discussion

This case illustrates an incidental diagnosis of complete ACC in late adulthood, consistent with the known association between ACC and intellectual disability [3]. While many individuals with ACC are diagnosed prenatally or in early childhood, those with milder phenotypes or baseline cognitive impairment may remain undiagnosed without neuroimaging. In some cases, individuals reach adulthood with relatively preserved functional status, and cognitive differences may be attributed to nonspecific developmental delay rather than an underlying structural abnormality [2,9]. This contributes to underrecognition of ACC outside of pediatric and subspecialty settings.

Current AAP guidelines recommend neuroimaging in children with intellectual disability only when additional features suggest underlying structural brain abnormalities, such as abnormal head circumference, focal neurologic deficits, seizures, or developmental regression [4]. As a result, individuals with isolated intellectual disability may not undergo imaging, potentially delaying or precluding diagnosis. In addition, access to specialty care, variability in developmental assessment, and differences in clinical thresholds for imaging may further limit early identification [4,10]. These gaps are reflected in adult populations, where incidental findings such as in this case may be the first point of recognition.

Radiographically, this case is consistent with prior descriptions of hallmark features of ACC including colpocephaly and characteristic ventricular configurations remain consistent across the lifespan. Recognition of these findings is important to avoid misinterpretation as hydrocephalus, particularly on initial CT imaging. Colpocephaly, disproportionate enlargement of the occipital horns, and parallel orientation of the lateral ventricles are well described and can be reliably identified on both CT and MRI [3,11]. Familiarity with these features is important in acute care settings, where ventricular enlargement may otherwise prompt unnecessary workup or intervention.

From a neurologic standpoint, outcomes in ACC are heterogeneous and depend on whether the anomaly is isolated or associated with additional malformations or genetic conditions [2,9]. Individuals with isolated ACC may have relatively preserved language and motor function but can demonstrate deficits in higher-order processing, including abstract reasoning, problem solving, and social cognition [2,9]. In this patient, cranial nerve examination was intact and bedside motor and sensory testing were unremarkable, consistent with the relatively preserved neurologic function described in isolated ACC. Formal neuropsychological evaluation was not performed, and therefore, higher-order cognitive domains including abstract reasoning, problem solving, and social cognition, could not be assessed in this case. Whether subtle deficits in these areas are present remains unknown, and this represents a limitation of the current report. These differences may persist into adulthood and influence occupational and social functioning, even in individuals who are otherwise independent.

Beyond clinical considerations, diagnosis of ACC in adulthood may have important social implications. Identification of a structural etiology for lifelong cognitive differences can provide validation, facilitate access to supportive services, and inform future care planning. In the literature, identification of a structural etiology for lifelong cognitive differences has been associated with validation of patient experience, facilitation of supportive services, and improved care planning [6,9]. A formal diagnosis may also guide anticipatory counseling, help contextualize prior educational and occupational challenges, and support connection to appropriate community resources [6,12]. In this case specifically, the diagnosis provided a clearer framework for understanding the patient’s developmental history, despite being incidental to the presenting complaint demonstrating how these broader implications can manifest at the individual level.

## Conclusions

ACC can remain undiagnosed into late adulthood in individuals with intellectual disability who have not undergone prior neuroimaging. This case highlights the importance of recognizing characteristic imaging findings to avoid misdiagnosis. In particular, ventricular enlargement on initial CT may be mistaken for hydrocephalus, which can lead to unnecessary consultations or interventions if classic features of ACC are not considered. Awareness of these radiographic patterns across imaging modalities allows for more accurate interpretation and appropriate clinical reassurance when findings are incidental. Establishing a diagnosis, even later in life, may also provide patients and caregivers with a clearer explanation for longstanding cognitive differences and may help guide supportive services. However, the practical impact of late diagnosis is likely to vary depending on individual circumstances, existing support systems, and the clinical context in which the diagnosis is made. This variability underscores the importance of individualized discussion with patients and caregivers when communicating incidental findings of this nature.
